# How Accurately Can Your Wrist Device Recognize Daily Activities and Detect Falls?

**DOI:** 10.3390/s16060800

**Published:** 2016-06-01

**Authors:** Martin Gjoreski, Hristijan Gjoreski, Mitja Luštrek, Matjaž Gams

**Affiliations:** Department of Intelligent Systems, Jožef Stefan International Postgraduate School, Jožef Stefan Institute, Ljubljana 1000, Slovenia; hristijan.gjoreski@ijs.si (H.G.); mitja.lustrek@ijs.si (M.L.); matjaz.gams@ijs.si (M.G.)

**Keywords:** activity recognition, fall detection, wrist, accelerometer, machine learning, classification, feature extraction

## Abstract

Although wearable accelerometers can successfully recognize activities and detect falls, their adoption in real life is low because users do not want to wear additional devices. A possible solution is an accelerometer inside a wrist device/smartwatch. However, wrist placement might perform poorly in terms of accuracy due to frequent random movements of the hand. In this paper we perform a thorough, large-scale evaluation of methods for activity recognition and fall detection on four datasets. On the first two we showed that the left wrist performs better compared to the dominant right one, and also better compared to the elbow and the chest, but worse compared to the ankle, knee and belt. On the third (Opportunity) dataset, our method outperformed the related work, indicating that our feature-preprocessing creates better input data. And finally, on a real-life unlabeled dataset the recognized activities captured the subject’s daily rhythm and activities. Our fall-detection method detected all of the fast falls and minimized the false positives, achieving 85% accuracy on the first dataset. Because the other datasets did not contain fall events, only false positives were evaluated, resulting in 9 for the second, 1 for the third and 15 for the real-life dataset (57 days data).

## 1. Introduction

Automatic recognition of daily activities and estimation of energy expenditure may assist with proper management of pathologies such as obesity, diabetes and cardiovascular diseases [1]. Moderate to vigorous physical activity is associated with decreased risk factors for obesity, cardiovascular and pulmonary diseases, cancer, depression, and increased bone health [2]. Accurate measurement of physical activity is therefore essential for developing intervention strategies and providing rich contextual information which can be used to infer additional useful information [3,4,5].

Beside normal activities, particularly older users also benefit from the detection of events such as falls. Falls are among the most critical health risks for the elderly [6]: approximately 30% of people over the age of 65 fall each year, and this proportion increases to 40% in those aged more than 70 [7]. About 20% of the elderly who fall require medical attention [8]. Furthermore, falls and the fear of falling are important reasons for nursing-home admission [9]. Falls are particularly critical when the elderly person is injured and cannot call for help. Automatic recognition of daily activities and detecting falls are therefore two of the most important tasks and represent basic building blocks in numerous health and telecare systems.

In recent years, wearable accelerometers have proven successful for recognizing activities and detecting falls [10,11,12], and are probably the most mature technology for this purpose. The reason for this is that they are capable of measuring human motion (mainly by measuring the linear 3D accelerations) and estimating body postures (mainly by measuring the orientation with respect to the Earth’s gravity). Multi-accelerometer systems have already shown the ability to recognize activities and detect falls with high accuracy [12,13]. However, having multiple sensors attached to the body is a burden to the user, which is probably the biggest reason why most multi-sensor systems are not well-accepted and are not commercially successful. Single-accelerometer systems are less accurate, yet they achieve good results when attached to the torso, which is generally considered the most suitable location [14]. However, many users dislike this placement, and thus such systems also enjoy limited success.

In contrast to dedicated activity-monitoring systems, wristband devices (e.g., FitBit-San Francisco, California, and Empatica-Milan, Italy) and smartwatches (e.g., Apple watch-San Francisco California, and Android wear wristwatches-San Francisco, CA, USA) are increasingly popular, mainly because people are accustomed to wearing watches, which makes the wrist placement one of the least intrusive placements to wear a device. However, developing a method for a wrist-worn device that will successfully recognize most of our daily activities and detect falls is quite a challenge. The reason for this is that the hand is usually the most active part of the body and produces more irregular movements compared to other body parts (e.g., the torso). There are some recent studies on this topic, however researchers usually find this placement less informative, achieving poor performance in activity recognition (AR) [11] and even more so in fall detection (FD) [15]. Single-wrist devices generally achieve lower fall-detection accuracy than devices attached to the torso, and suffer from a high false-positive rate.

In this paper we compare the performance of AR and fall detection with accelerometers positioned at various locations on the body on three datasets. Our objective is to quantify how much worse the wrist performs compared to other locations, if it indeed is worse. To do so, we first developed methods for AR and FD, and then systematically evaluated them on three datasets that included accelerometers placed on at least one wrist. The datasets were recorded by 21 volunteers, resulting in 195 h of multi-sensor labeled data (of which 47 h are wrist data). We then evaluated our methods on further 1367 h of unlabeled wrist data recorded in unconstrained real life. A recent study by Mannini *et al.* [16] showed that their dataset is significantly larger compared to 5 others and includes 1609 min of wrist data, which is significantly less compared to ours 2820 min of labeled wrist data.

As an additional objective, we studied how the left (in our datasets the non-dominant) wrist compares to the right (in our datasets the dominant) one. Also we performed a “switching wrists” analysis, *i.e.*, we compared various combinations of training and test data recorded on the left, right or both wrists.

The rest of the paper is organized as follows. In the next subsection we describe the most relevant related work. Then, in Section 2 we describe the sensors and the data collection. Section 3 describes the methods developed for the AR and FD. Section 4 describes the results and provides related discussion. Section 5 provides a discussion about the left vs right wrist paradigm. Finally, Section 6 provides the conclusion and the remarks for future work.

### Related Work

The most recent literature in the AR field shows that wearable accelerometers are among the most suitable sensors for unobtrusive AR [17]. Accelerometers are becoming increasingly common due to lowering cost weight and power consumption. Currently the most exploited and probably the most mature approach to AR is using machine learning (ML) methods on wearable accelerometer data [11,18,19]. This approach usually implements widely used classification methods, such as Decision Tree, Random Forest, Support Vector Machines, K-Nearest Neighbors, Naive Bayes, and recently deep neural networks [20,21].

For the sake of the user’s convenience, AR applications are often limited to a single accelerometer, even though nearly all reports find that better performance is achieved with more accelerometers. Numerous studies have shown that the performance of AR systems strongly depends on accelerometer placement (e.g., chest, abdomen, waist, thigh, ankle) and that some placements turn out to be more suitable (in terms of AR performance) for particular activities [10,11,12].

In the past the wrist was the least exploited placement for AR, mainly due to frequent hand movements which negatively influence an AR system [11,22]. The researchers were usually testing chest, waist, thighs (left and right) [18,23], ankles (left and right) [24] and neck [25]. For example, a recent overview of AR systems showed that only 5 out of 13 analyzed systems included wrist data in their systems [11]. The results vary a lot and cannot be compared to different studies (different datasets, different algorithm parameters, different approaches, *etc.*). In our previous work we also tested most of these locations on two datasets. On the first one, the results showed that all of the locations perform similarly achieving around 82% accuracy [24]. On the second dataset, where the experiments were more thorough (bigger dataset, improved algorithms) the results showed that thigh and ankle perform similarly (82% and 83% respectively) and achieve higher accuracy compared to the chest (67%) [26].

However, with the penetration of the wrist-worn fitness trackers and smartwatches the wrist sensor placement is becoming a matter of interest. Recently, Trust *et al.* [27] presented a study for hip *versus* wrist data for AR. The models using hip data slightly outperformed the wrist-data models. Similarly, in the study by Rosenberg *et al.* [28] for detecting sedentary behavior, the models using hip data outperformed the wrist models. In the study by Manini *et al.* [16] ankle data models achieved high accuracy of 95.0% that decreased to 84.7% for wrist data models. Ellis *et al*. [29] presented an approach for the recognition of locomotion and household activities in a lab setting. For one subset of activities the hip-data models outperformed the wrist data, but over all activities the wrist-data models produced better results. Our study confirms that wrist performs worse than ankle, knee, and belt, but it is better than elbow and chest.

Garcia-Ceja *et al.* [30] presented person-specific wristband AR for activities such as: shopping, showering, dinner, computer-work, and exercise. Similarly, Attal *et al.* [31] used 10-fold-cross validation to evaluate their models and additionally used 1 second window of data with 80% overlap which results in having similar instances in the training and evaluation dataset. This explains the high accuracy (99%). In our study, we are not just analyzing general models (using leave-one-subject-out evaluation technique), we are also comparing laboratory data with the data gathered in the wild (how good a model build on laboratory data can perform in real life) by testing our models on four different datasets, out of which one is completely acquired in real life.

Another problem that we investigate is right *vs*. left wrist AR. Dieu *et al.* [32] performed similar analysis using ActiGraph which outputs counts, but not granular activities. They have found that there is no significant difference in the activity level estimation (number of counts) between the dominant and non-dominant hand. They showed that the count output of the ActiGraph sensor is highly correlated for both wrists. However, in this study the authors only used the count output of the ActiGraph sensor, which is a rough estimate of the activity intensity and not the particular activity of the user (e.g., sitting, standing, lying, walking, *etc.*). In our study we thoroughly investigated this issue by first creating a ML model that recognizes activities of the user, then compared the AR performance of both left and right wrist sensors, and finally we investigated the AR performance of applying left-wrist AR model on the data provided by the right wrist sensor, and *vice versa*.

## 2. Sensors and Data Collection

We performed our analysis on four independent datasets with the same preprocessing and feature extraction. We compared five machine-learning algorithms on each dataset and included the best one in the final analysis. We believe this strikes a balance between tuning our approach to each dataset, which may yield results that do not generalize to other datasets, and using a completely generic approach, which performs worse than a typical approach to AR and FD (which is adapted to its particular problem). The first dataset was recorded in our Ambient Intelligence Laboratory at Jožef Stefan Institute by five participants. The second dataset was recorded at the Faculty of Sports (FoS) in Ljubljana, Slovenia by ten participants. The third dataset is the Opportunity dataset [33,34], which is the most commonly used benchmark dataset for AR. Finally, the last dataset was recorded in real-life conditions (and therefore was not labeled with activities) by three participants who were wearing a wrist device for several weeks. Informed consent was obtained from the participants prior to participation for each dataset recorded in our laboratories. Details about the datasets are shown in Table 1. The datasets recorded in our laboratories will be available online in our AmI repository [35].

Each of the datasets contains different activities and was recorded by different types of accelerometers, which makes it difficult to directly compare AR and FD across datasets. However, all the datasets contain typical every-day life activities and all the accelerometers are of adequate quality for AR and FD, so we expect the findings within each dataset to be valid for other tasks dealing with everyday life and common accelerometers.

### 2.1. JSI Dataset

For the first dataset, a 120-min scenario was designed in cooperation with a medical expert to capture the real-life conditions of a person’s behavior, although it was recorded in a laboratory. The scenario was performed by five healthy volunteers (29.4 years, SD = 2.1, 4 males and 1 female) and included ten elementary activities (the percentage of instances per class): cycling (18%), walking (17%), standing (14%), lying (13%), sitting (11%), running (9%), on all fours (7%), kneeling (5), bending (3.5), and transition (2.5%). These activities were selected because they are the most common elementary everyday life activities. They were grouped into three groups. The first group were exercise activities: walking, running, and cycling (each around 10 min). In the second group, elementary activities and transitions between the activities were recorded. The sequence of activities performed in these sub-scenarios was predefined and volunteers were asked to follow them. Additionally, there was a specially designed sub-scenario that included two falls (tripping and slow fall) and fall-like events (quickly sitting down and quickly lying on the bed), which was repeated three times by each volunteer. In the third group, everyday life activities were recorded. The sequence of activities was not predefined and the volunteers were asked to mimic their normal, everyday life behavior when executing activities such as cooking, reading, typing, washing dishes, scrubbing the floor, *etc*.

**Figure 1:**
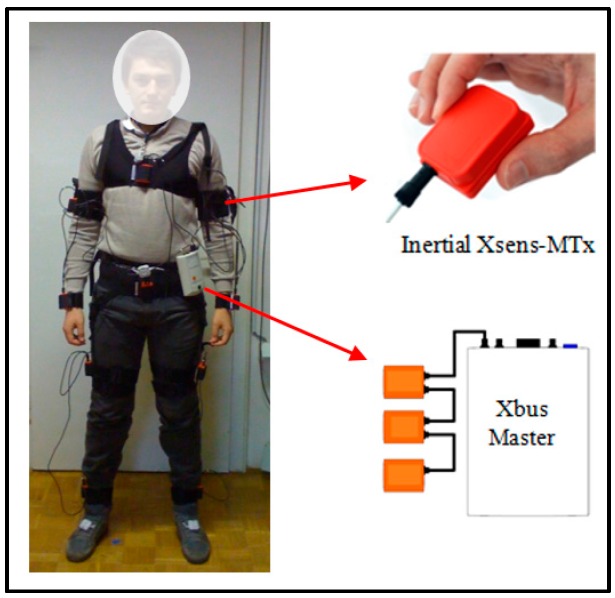
Xsens sensor placement.

The sensor equipment included ten Xsens-MTx inertial sensors (MTx-49A53G25). They were placed on the chest, waist, left and right thigh, left and right ankle, left and right upper arm, and left and right wrist (see Figure 1). The sensors were connected to a wearable central unit (Xbus Master), which transmitted their data to a laptop using Bluetooth. The accelerometer’s sampling frequency was set to 50 Hz (the maximum is 512 Hz), and the sensitivity was set to ±4 g. The labeling of the data was performed by a supervisor, who labeled each activity in real-time.

### 2.2. Faculty of Sports Dataset

The second dataset, was recorded at the Faculty of Sports in Ljubljana, Slovenia. The scenario was similar to the first dataset, except that the fall sub-scenario was omitted and it was focused more on the exercise activities. In particular the walking activity was performed on a treadmill with a one-percent inclination at 4 and 6 km/h, the running activity was also performed on the treadmill with a one-percent inclination at 8 km/h, and the cycling activity was performed on a stationary bicycle with 65 RPM with the difficulty set to 80 watts for the first six minutes and 160 watts for the other six minutes. The scenario was performed by ten healthy volunteers (age 27.2 years, SD = 3.1, 8 males, 2 females), and included the same eight elementary activities as in the first dataset (the percentage of instances per class): lying (23%), standing (17%), walking (14%), sitting (12%), cycling (10), on all fours (8%), kneeling (6%), running (5%), bending (2%), and transition (3%).

**Figure 2:**
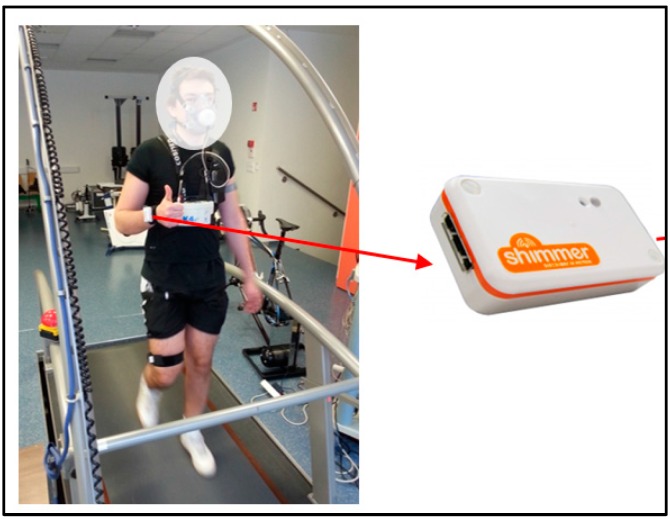
Shimmer data acquisition.

The sensor equipment included ten Shimmer accelerometer sensors (Shimmer 2R, Realtime Technologies). The sensor beside the accelerometer has an on-board MSP430 microcontroller, wireless communication via Bluetooth or 802.15.4 low power radio, and an option of local storage to a 2 GB micro SD card. It additionally provides digital (I2C, SPI) communication for new potential sensors. The 450 mAh Li-ion battery lasts for 8–10 h when the sensor sends the data in real-time through Bluetooth. To record the user’s acceleration, 50 Hz data sampling frequency was used and acceleration sensitivity level of ±4 g. This sensitivity level and sampling frequency were chosen based on empirical analysis of the human movements in our previous tests. It was shown that it is a reasonable tradeoff between the quality of the data on one side and the sensors’ battery consumption on the other side. The sensors were placed on the chest, thigh, ankle, and right wrist with adjustable straps (Figure 2). The data was acquired on a laptop in real-time via Bluetooth. The data was again manually labeled with activities.

### 2.3. Opportunity Dataset

The Opportunity AR dataset is a benchmark commonly used dataset for AR. It contains human activities related to a breakfast scenario, which are captured by sensors configured on three subjects who perform everyday life activities. There are four classes in this AR task: standing (50%), walking (28%), sitting (19%), and lying (3%). Each subject performed six runs, five are activities of daily living runs characterized by a natural execution of daily activities, and the sixth run is a "drill" run, where users execute a scripted sequence of activities (open/close fridge, open/close dishwasher, open/close drawers, open/close door, clean the table, drink, *etc*.) and has 20 repetitions.

The sensors include a wide variety of body-worn, object-based, and ambient sensors—72 in total. However, for the need of our study we only used the acceleration data from left and right wrist (InertiaCube3 inertial sensors). The sampling rate of the sensor signals is 30 Hz, and the data was acquired though Bluetooth.

### 2.4. Real-Life—Empatica Dataset

The last dataset was recorded in real-life conditions (*i.e*., a wristband device was given to three healthy male participants (age 24, 28 and 34) to wear it on the left wrist for several weeks each). The device was worn during the whole day, except for 1–2 h when it was charging the battery.

We used the Empatica E3 wristband [36] and logged the accelerometer data in the internal memory with 32 Hz accelerometer sampling frequency. This data was not labeled, therefore it could not be used to calculate the performance of the AR method. However, we used a model trained on the first dataset to recognize the activities for this dataset. This way we were able to provide an overview of the (recognized) activities for each person during several weeks. Similarly, for the FD we used the model trained on the first dataset and evaluated it for false positives (because there was no fall during the recordings).

## 3. Methods

### 3.1. Activity Recognition

**Figure 3:**
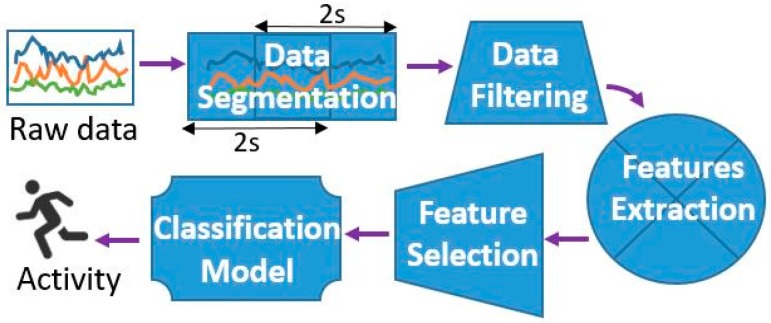
Activity recognition approach.

Figure 3 shows the ML approach used in this research. It includes the following modules: data segmentation, data filtering, feature extraction, feature selection, and building a classification model. This approach has been refined over several years of experimenting on various domains, resulting among others in the first place at the EvAAL AR and FD competition [17].

Note that, all of the parameters (e.g., choosing the subset of features by feature selection) were tuned on the first dataset. Later, the same experimental setup was used for each of the other datasets to avoid, the problem of tuning parameters to a particular dataset.

#### 3.1.1. Data Segmentation, Filtering, and Feature Extraction

The data segmentation phase uses an overlapping sliding-window technique, dividing the continuous sensor-stream data into data segments—windows. A window of a fixed size (width) moves across the stream of data. We used 4 s windows with 2 s overlap, which was defined empirically in our previous work [37]. Once the sensor measurements are segmented, further pre-processing is performed using two simple filters: low-pass and band-pass. The feature extraction phase produces 52 features (summarized in Table 2) from the accelerations along the x, y, and z axes. The first seven features (Mean X/Y/Z, Total mean, and Area X/Y/Z) provide information about body posture, and the remaining features represent motion shape, motion variation, and motion similarity (correlation). More thorough analysis of the features can be found in Tapia’s PhD thesis [38].

#### 3.1.2. Feature Selection

Since all of the features are extracted from one data source (wrist accelerometer), a high feature correlation is expected. For that reason the feature selection method is based on feature-correlation analysis, which removes correlated and “non-informative” features. Non-informative features are considered those that have a low information gain. The information gain evaluates the worth of a feature by measuring the information they carry about the class. Regarding the correlation of the features, we checked for Pearson’s correlation, which measures linear correlation between features, and Spearman correlation, which measures how well the relationship between two variables can be described using a monotonic function. The feature selection steps are:

- Rank features by gain ratio.
- Starting from the lowest ranked feature, calculate its correlation coefficients (Pearson and Spearman) with each of the features ranked above. If it has a correlation coefficient higher than 0.80 with at least one feature, remove it.
- Repeat step 2 until 70% of the features are checked.

**Figure 4:**
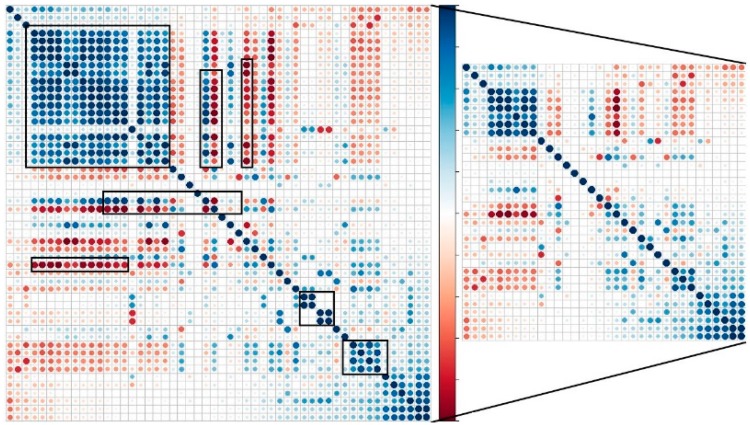
Person’s correlation matrix before (**Left**) and after (**Right**) feature selection.

Figure 4 shows the results of the Person’s correlation analysis before (left) and after (right) the feature selection phase. On the figure there are two correlation matrices, 52 × 52 (left) and 34 × 34 (right). Each row (column) represents a different feature. Red color represents negative, blue color represents positive, and the intensity of the color represents the absolute value of the correlation. This figure, on one hand, depicts the dimensionality reduction of 37% (from 52 features to 33 features), and on the other hand the correlation reduction (the intensities of the colors). On the left matrix some regions with high correlation are marked (with black rectangles) to present candidate features that the feature selection algorithm may delete. On the right matrix there is high correlation between some of the features even after the feature selection phase. These are features that have high gain ratio. In each experiment we checked the accuracy with and without the feature selection phase. The experiments with feature selection phase achieved at least equal results and in some cases slightly better results with a lower computational complexity, since feature extraction is computationally the most demanding part of AR. This is very important for AR on a smartphone or on a sensor node itself.

#### 3.1.3. Classification

Once the features are extracted (and selected), a feature vector is formed. During training, features vectors extracted from training data are used by a ML algorithm to build an AR model. During classification, feature vectors extracted from test data are fed into the model, which recognizes the activity of the user. We compared several ML algorithms: Decision Tree (J48) [39], Random Forest (RF) [40], Naive Bayes (NB) [41], Support Vector Machines (SVM) [42], and K-Nearest Neighbors (KNN) [43].

A decision tree is a predictive model which maps observations about an item (feature values) to conclusions about the item’s target value. A tree is built by splitting the training dataset into subsets based on an attribute value test. In these tree structures, leaves represent class labels and branches represent conjunctions of feature-values.

RF is an ensemble classifier which constructs multiple Decision Trees (10 by default or 100 in some newer version of the WEKA toolkit) and outputs the majority class label from the constructed trees. It utilizes a random step in the process of creating the trees and selecting a splitting feature which makes it robust to noise and outliers.

NB classifier is a simple probabilistic classifier based on applying Bayes' theorem with strong (naive) independence assumptions. It assumes the conditional independence of the feature which makes it inferior in the case of AR using acceleration data since there is high correlation between the features.

KNN is a distance based classifier which doesn’t learn a model, instead, it calculates distance (Euclidean by default) from a new instance to each instance in the training dataset and it assigns the new instance to the majority class label from the closest k neighboring instances in the training dataset.

SVM is a binary classifier which uses statistical learning theory to provide hyperplane in the feature space that divides the instances according to the class value (in our case activities). It uses a kernel function to deal with non-linearly separable data and classification tricks such as “one-*versus*-all” or “pairwise classification” to deal with multiclass problems.

### 3.2. Fall Detection

Wearable accelerometers are probably the most exploited sensors for fall detection mainly because they can measure the free fall of the body, the impact, and the lying that typically follows a fall. Because of this, the accelerometer is usually fixed on the parts of the body that do not have large accelerations during everyday activities, such as the user’s torso (chest, abdomen, and waist). The wrist is one of the placements to avoid, because it results in large acceleration during everyday activities and therefore gives lots of false alarms [15]. However, in our experiments we use the assumption that after a fall, the person is not moving or moving very little. This way we reduce most of the false alarms that occur during other activities. Of course this is quite a strong assumption, but the justification is that if the person is moving after the fall, that he/she is conscious enough to call for help. Therefore, our method has two modules: the first one detects an acceleration fall pattern (AFP) and the second one checks how much the person is moving. Both are described in the following sub-sections.

#### 3.2.1. Acceleration Fall Pattern (AFP)

**Figure 5:**
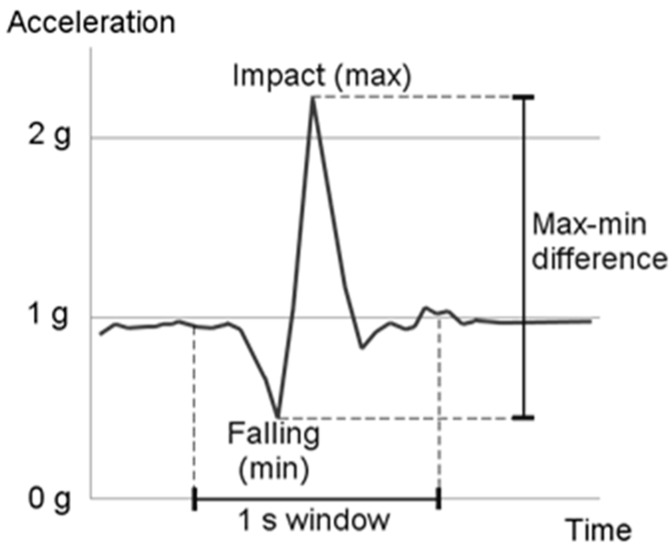
Example acceleration data for fall detection.

As the name suggests, the AFP constantly monitors the acceleration signal (the module of the acceleration signal) and detects a fall pattern. The rationale for this method is that the acceleration pattern during a typical fast uncontrolled fall (shown in Figure 5) shows a decrease in the acceleration (free fall) followed by a rapid increase (impact with the ground). In our implementation of the AFP, the difference between the maximum and minimum accelerations within a one-second window was calculated. If the difference exceeded the threshold and the maximum appeared after the minimum, a fall was declared. The threshold was chosen empirically based on preliminary data [12] and was set to 21 m/s^2^.

#### 3.2.2. Movement Detection

During motion, accelerometers produce a changing acceleration signal, and the more intense the motion, the greater the change in the signal. Using these changes, the Acceleration Vector Changes (AVC) feature was extracted [14]. This feature sums up the differences between consecutive values of the lengths of the acceleration vector, and divides the sum by the time interval (one second):

(1) $$AVC=\frac{\sum_{i=1}^{n}|length_{i}-length_{i-1}|}{T_{n}-T_{0}}$$

*T*_0_ is the time stamp for the first data sample in the window, and *T*_n_ is the time stamp of the last data sample. The *length* is the length (module) of the acceleration vector along the x, y, and z axes ($a_{x}$, $a_{y}$, and $a_{z}$, respectively), calculated as:

(2) $$length=\sqrt{a_{x}^{2}+a_{y}^{2}+a_{z}^{2}}$$

By applying a threshold to the AVC value, the movement of a sensor is detected. The threshold was chosen empirically and was set to *n* * 0.7.

Even though our fall detection approach is relatively simple, it substantially outperformed a more sophisticated machine-learning approach [44], and it has the added advantage of low computational complexity.

## 4. Experimental Results

### 4.1. Activity Recognition

For the evaluation of the AR, the leave-one-person-out cross-validation technique was used. This means that the model was trained on the whole data set except for one person on which it was later tested. This procedure was repeated for each person. This evaluation approach is more reliable than using the same person’s data for training and testing, which gives overly optimistic results. Four evaluation metrics commonly used in AR were analyzed: the recall, precision, accuracy, and F-measure (F1).

We also compared the performance of five ML algorithms on each dataset: J48, SVM, NB, RF, and KNN. Except for the KNN algorithm where the parameter k was set to be five, all of the algorithms were used with their default parameters as implemented in the WEKA ML toolkit. For the feature selection, we used the previously described feature selection algorithm on a sub-dataset which consists of 10% of the (randomly selected) instances of each of the five subjects. The feature selection algorithm managed to reduce our feature set from 52 to 33 while keeping the accuracy on the same level.

#### 4.1.1. JSI Dataset

**Figure 6:**
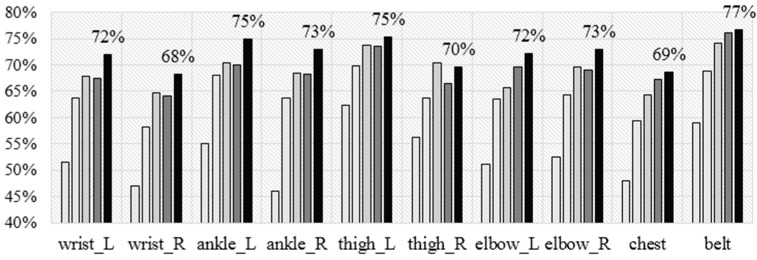
Accuracy for wrist *vs.* other sensor placement for the activity recognition (AR) on the Jožef Stefan Institute (JSI) dataset.

Figure 6 shows the accuracy achieved by each of the five algorithms for each of the sensor placements using the leave-one subject-out (LOSO) cross validation technique. In general NB performs the worst, J48 is slightly better, next are SVM and KNN, which perform similarly, and RF performs best. The maximum accuracy is 77%, which is achieved by RF for the belt sensor. These results are in line with the study by Cleland *et al.* [11], which also showed that SVM outperforms J48 and NB in almost any sensor location (they had six), however, they have not tested the RF, which performs best in our experiments. Additionally, in their experiments, chest is the best performing placement compared to the left wrist, thigh, and ankle; which is not in line with our experiments (*i.e*., we showed the chest achieves the lowest accuracy (69%) compared to the left wrist (72%), left thigh (75%), and left ankle (75%)). Because we had sensors on both wrists, we compared the accuracies and noted that the left wrist is better compared to the right one (72% *vs*. 68%). Also, the left wrist is better compared to the elbow and the chest, and worse compared to the ankle, knee, and the belt.

Table 3 shows the confusion matrix, precision, recall, and F1 score for each class obtained by the RF classifier using the data of the left wrist. Data in the confusion matrix is expressed in fractions (percentages) with respect to the overall number of instances with the specific class label shown in the column “overall #”. The F1 score shows that kneeling and transition are the two activities that are the hardest to recognize for the classifier. This should not be a large problem for most applications though. Standing, sitting, bending, and all fours are recognized moderately well, probably because they are distinguished by body postures, which are not characterized well by wrist orientation and motion. Walking, lying, cycling, and running are the activities that are recognized on a satisfying level, because they have distinctive (wrist) motion and/or orientation.

#### 4.1.2. Faculty of Sports Dataset (FoS Dataset)

**Figure 7:**
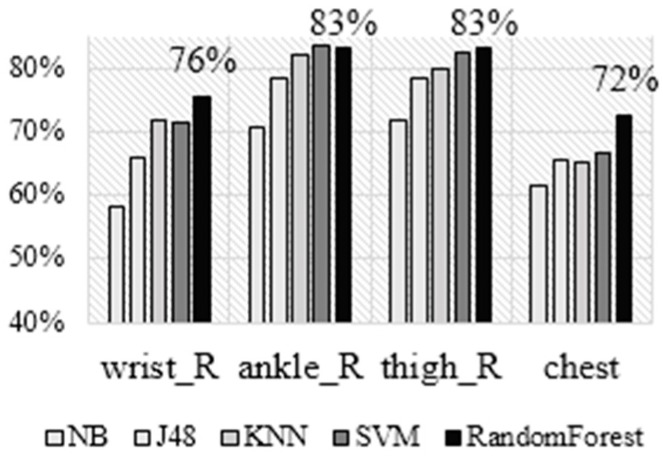
Accuracy for wrist vs other sensor placements for the FoS dataset.

The results for the second dataset are presented in Figure 7 showing the accuracy achieved by each of the five algorithms for each of the sensor placements using the LOSO cross validation technique. In general, our results are consistent with the results obtained on the JSI dataset. Namely, NB performs the worst, J48 is slightly better, next are SVM and KNN, which perform similarly, and RF again performs best. Regarding the sensor placements, by looking at the best performing classifier (RF) we can note that the ankle and the thigh achieve the best accuracy, followed by the wrist and the chest. In terms of absolute numbers, the accuracies are slightly higher compared to the ones achieved on the JSI dataset: the accuracy for the right wrist is 76%, which is higher than the 68% achieved on the JSI dataset; the difference is even larger for the ankle and thigh, but smaller for the chest. A possible reason for this may be that in the second dataset there is more training data (nine subjects are used for training compared to four in the first dataset). The advantage of the best two placements over the wrist is similar to the one in the JSI dataset, but it should be noted that only the right wrist is available in this dataset, which is less suitable for AR than the left one.

Table 4 shows the confusion matrix, precision, recall, and F1 score for each class obtained by the RF classifier using the data wrist data. Similar to the previous confusion matrix, the data is expressed in fractions (a percentage) of the overall number of instances with the specific class label shown in the column “overall #”. As with the JSI data, kneeling and transition are the two activities that are the most difficult to recognize, bending, and standing are still in the middle, whereas sitting and all fours joined the activities walking, lying, cycling, and running as activities that are recognized on a satisfying level. The improvement of the F1 scores and the overall accuracy is probably due to a larger number of training instances. Our training data was acquired by nine people, whereas the JSI dataset only includes four people.

#### 4.1.3. Opportunity Dataset

**Figure 8:**
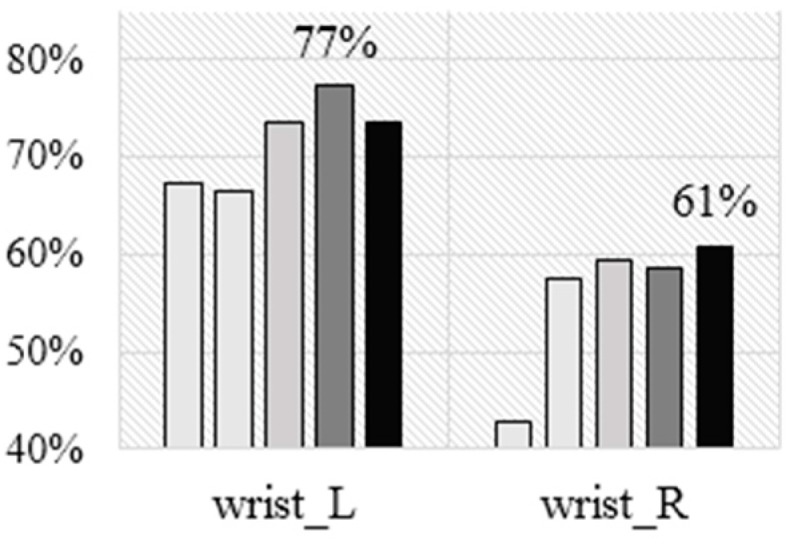
Accuracy for left vs right wrist for the Opportunity dataset.

Next, we tested the AR method on the Opportunity dataset. Figure 8 shows the accuracy for a four class problem (standing, walking, sitting, and lying) achieved by each of the five algorithms for left and right wrist placements, using the LOSO cross validation technique. Similarly to the results on the previous datasets, NB performs the worst, J48 is slightly better, next is KNN, SVM is best on the left wrist, and RF is best on the right one. Overall, RF seems to be the most suitable ML algorithm for AR, even though it is not the best in every single case.

The accuracy of the left wrist is better than on the JSI dataset, although it may be lower than on the Faculty of Sports dataset if it was available there. The accuracy of the right wrist is worse than in either of the previous two datasets. This may be because the dataset contains breakfast and cooking activities, where the subjects use their right hand a lot. This suggests that while the left wrist is almost always preferable, the right one may be acceptable in some applications but not in others.

Table 5 presents the confusion matrix of the LOSO cross validation using the left wrist data in which we report fractions from the overall number of instances with the specific class label shown in the column “overall #”. It can be noticed that on one hand the classifier is mixing the activities standing and walking, and on the other hand sitting and lying. This is in line with the results from the experiments on the previous two datasets.

Even though Opportunity is a benchmark dataset for AR, we have managed to find only one study that performs AR with the wrist sensors, by Nguyen *et al.* [45]. Because they are reporting the accuracy using 10-fold cross validation, we additionally performed experiments using this technique. Our method achieved accuracy over 85% for each subject, and the left wrist performed slightly better compared to the right wrist. The highest accuracy achieved was 95% for the data of the subject 1. Additionally, our approach outperformed the RF that was used by Nguyen *et al.* [45] (*i.e*., our RF achieved 90% average accuracy which was by 4 percentage points better than theirs). This shows the advantage of our feature pre-processing techniques (feature extraction and feature selection).

#### 4.1.4. Real-Life Empatica

Finally, we applied our AR system on real-life data. The AR model was trained on the JSI dataset, since the real-life data was not completely labelled. The only labels available to us were sleeping events, labeled by the subjects, and for these events we can be sure that the subjects are lying. Table 6 presents the percentages of recognized lying instances for the overall dataset. The classifier recognized 66% of the 534,000 lying instances (nearly 300 h of sleeping). It can be noticed that that the classifier is mixing the classes lying and sitting which again is in line with the experimental results on the previous three datasets.

For the rest of the real-data, evaluation metrics cannot be reported, however, we will summarize the predictions of the classifier. Table 7 presents per-person results of the AR system. It reflects the typical sedentary behavior of a computer science researcher, which is the profile of the three subjects. Due to the sensor’s recommendations (the Empatica E3 sensor is not completely waterproof), the sensor was usually not used during exercise, so the number of running instances is quite low, as expected.

**Figure 9:**
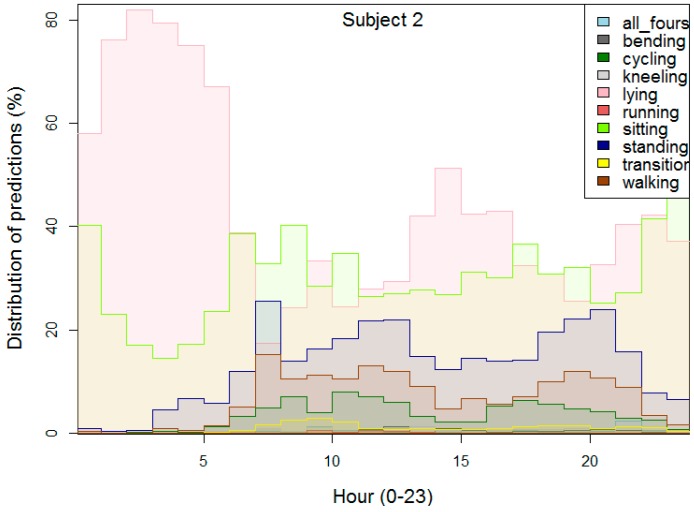
Distribution of the predictions (recognized activities) of a subject, per hour for the real-life dataset.

Figure 9 depict the distribution of the recognized activities for one of the subjects. On the x-axis is the hour of the day, and on the y-axis is the percentage of predictions (recognized activities) for a certain hour calculated over the whole dataset for the subject. For example, it shows that from 2 am to 3 am, roughly 15% of the activities are sitting and 85% are lying. Additionally, it can be noticed that with activities like “sitting” the subjects are probably sleeping (“lying”) (e.g., from 1 am to 5 am) and *vice versa*, “lying” subjects are probably sitting (working on a computer), e.g., from 10 pm until 5 pm. The mixing of the two classes “lying” and “sitting” was already confirmed by the experiments on the first two datasets. A simple solution for this problem would be to add one feature that reflects the hour of the day, but again it depends on the application of the AR system.

After showing these types of figures to the three subjects, they all agreed that the figures more or less depict their daily routine. For example, the subject that is shown in Figure 9 confirmed that he typically goes to sleep at 11 pm and rises around 6 am. The recognized activities suggest his sleep is shorter, which may be due to movement at the beginning and end of the sleeping period. He sometimes takes an afternoon nap during the weekend, which can also be seen as lying. He is very active around 8 am when he takes children to kindergarten and then goes to work, which can be seen as a spike in walking and standing. The comparable amount of sitting and standing around noon (when he goes for lunch) and in the evening is a bit surprising, since he feels he is more active in the evening.

### 4.2. Fall Detection

To evaluate the FD, one must decide how to weigh the undetected falls and the false alarms. Both are important: not detecting a fall may endanger a person's health, while false alarms make the system unlikely to be used in real life. Therefore, we present the True Positive Rate (TPR), the True Negative Rate (TNR), and accuracy. TPR is the fraction of correctly recognized falls out of all falls. TNR is the fraction of correctly recognized non-falls out of all non-falls.

#### 4.2.1. JSI Dataset

To evaluate the FD algorithm on this dataset, we first divided the activity scenario into events. For example, the person sitting on the chair is one event which includes dozens of seconds of activities (walking, sitting down, and sitting). This way we were able to divide the activity scenario into 130 events in total, 30 of which were fall events (15 fast falls and 15 slow, controlled falls) and 100 were non-fall events, among which 30 were designed to resemble falls (15 quickly sitting down and 15 quickly lying on the bed). We first compared the different sensor placements for FD. The results are shown in Table 8. The left wrist proved to be the best location, which is contrary to the conventional wisdom, which claims that the torso is the best location for FD [22,23]. This can be explained by the fact that during slow falls the torso experiences very little acceleration, which is not always the case for the wrist, since it is more mobile. The left wrist is again preferable to the right because the right one triggers more false positives.

Table 9 (on the left) shows the confusion matrix for the FD method for the left wrist. It shows that the method detected 21 of the 30 falls (TPR = 70%) and it detected 10 false falls during the 100 non-fall events (TNR = 90%). Additional analysis showed that all of the fast falls (e.g., tripping) are detected and that 40% of the slow falls are detected. Given the fact that the method uses the high acceleration (AFP) as a trigger, this is a promising result. Next, the analysis showed that most of the false positives (8 out of 10) occur during the event of quickly lying down (jumping into bed). This was also expected because the acceleration is high enough to trigger the AFP and the person is lying without movement afterwards. Finally, we checked the performance of the AFP only (without the movement analysis), which is a commonly used method in the literature [46]. The results show that the 21 falls that are triggered by the AFP are actually recognized by the advanced method (*i.e*., the movement analysis did not disregard any of the true positives). The only problem with the AFP is that it produces too many false positives (*i.e*., it falsely detected 63 events as falls out of 100).

The confusion matrices for the right wrist show similar results (Table 10). They show that the right wrist performed worse compared to the left (*i.e*., detected 17 of the 30 falls (TPR = 57%) and it detected 13 false falls during the 100 non-fall events (TNR = 87%)). Also, in the case of the right wrist, two fast falls which were detected by the AFP were discarded by the advanced method.

#### 4.2.2. Faculty of Sports Dataset

The second dataset, Faculty of Sports, did not contain fall events, therefore the evaluation of the FD method was only for false positives (detected falls during a non-fall event). The results showed that chest and right thigh do not generate a lot of false positives (one and two, respectively) compared to the right wrist (nine false positives) and the right ankle (16 false positives).

These results are quite promising if we take into account that the dataset consists of 10 subjects (102 h of data) and that only nine false positives were detected with the wrist sensor. Additional analysis showed that six of the false positives occurred during a lying event, which is similar event to a typical fall when the person is not moving after a high acceleration. Thus, if there are high accelerations produced by the hand when the person changes sides while sleeping, followed by inactivity, FD recognizes this as a fall. It may be possible to address this by switching into a “resting” mode when a prolonged period with little movement is detected without a high acceleration at the beginning. In this experiment the chest did prove the best placement as the conventional wisdom suggests. However, one should not forget that this dataset did not contain any falls, where the wrist may prove as good or better (as was the case for the JSI dataset).

#### 4.2.3. Opportunity Dataset

Similar to the second dataset, the Opportunity dataset also did not contain fall events, therefore we tested the method for false positives. The results showed that only one false positive on the right wrist was detected while the person was standing.

#### 4.2.4. Empatica Dataset

Finally, we applied the FD method to the real-life Empatica dataset, which also did not contain any fall events. The results showed that 15 false alarms were detected in total. Given the fact that the dataset contains 1378 h (more than 57 days) of acceleration data recorded in real life, this is quite an encouraging result. By using the timestamp when the false alarm happened, we additionally analyzed the false alarms. We first discovered that four of them happened when the user was taking the wristband off (e.g., in order to charge it). This may be easily solved by simply having a sensor that will detect when the wristband is worn or not (some of the current wristbands already have this feature). Additionally, we discovered that most of false alarms (seven out of 13) occurred when the person was sleeping. This matches the findings in the Faculty of Sports dataset. It may be a problem in practical applications, and should be addressed in the future, especially since the elderly often fall when getting up at night. A possible solution is again switching into a “resting” mode when the person goes to sleep, and switching into the normal mode by detecting prior movement before a potential night fall, to recognize that the person rose from the bed. However, such an approach would not be able to detect falls from the bed. The last four false alarms happened when the subjects were at work. Our results compare favorably to a systematic comparison of 13 fall-detection methods [47], which raised between three and 85 false alarms in 24 h.

## 5. Left (Non-Dominant) *vs.* Right (Dominant) Wrist Discussion

In general, our experiments showed that the left wrist performs better compared to the right wrist in both the AR and FD tasks. Since all the measured individuals are right-handed (except one in the real-life dataset) it means that the wrist on the non-dominant hand is more informative. However, compared to laboratory experiments, real-life poses additional challenges that should be taken into account. A situation that may occur in real life is the wristband being worn on either of the wrists. It might happen that the model is developed for the left wrist and the user wears the device on the right wrist, and the person can be indiscriminately left- or right-handed. The experiment was performed in such a way that we trained the model on the data of one of the wrists and tested it on the data of the other wrist. Additionally we created a model using the data from both wrists and tested it on each wrist individually. The experiment was performed on the first dataset because it contains data for both wrists.

Table 11 shows the recognition rate (recall) for each of the 10 activities and the overall accuracy for four combinations: (i) training on the left wrist and testing on the left wrist (Left-Left), (ii) training on the right wrist and testing on the right wrist (Right-Right); (iii) training on the left wrist and testing on the right wrist (Left-Right); and (iv) training on the right wrist and testing on the left wrist (Right-Left). The results show that the Left-Right and the Right-Left combinations achieve 55% and 53% accuracy, respectively, which is significantly lower compared to the Left-Left (72%) and Right-Right (68%). The recall values for each of the activities show that the biggest drop in recall is for the: standing, sitting, and running activity. The detailed analysis of the confusion matrix showed that running is recognized mostly as standing and that sitting is mostly recognized as lying. Standing is misrecognized as multiple activities including: walking, sitting, lying, bending, transition, and on all fours.

Additionally, we tested two combinations which include the data from both wrists for training and testing on each of them individually (*i.e*., (i) training on both the right and the left wrist and testing on the left wrist ((Right + Left)-Left), and (ii) training on both the right and the left wrist and testing on the right wrist ((Right + Left)-Right)). This way, we were able to increase the accuracy and achieve not only better accuracy compared to the Left-Right and Right-Left combinations, but also better compared to the classical Left-Left and Right-Right. Therefore, to solve the problem of switching the wrists, one should deploy a model trained on both hands.

## 6. Conclusions

Motivated by the recent popularity of wristband devices and smartwatches on one side, and the challenge to create an accurate method for AR and FD on the other side, we performed a systematic evaluation of our methods for AR (ML) and fall detection (domain knowledge encoded with expert rules) on four datasets (two laboratory datasets, the Opportunity dataset, and a real-life dataset). The methods were evaluated on 21 volunteers in total, resulting in 195 h of multi-sensor labeled data (of which 47 h are wrist data) and 1367 h of wrist unlabeled data. This is, to the best of our knowledge, the largest evaluation of AR and FD using wristband accelerometers.

Since our targets are battery-powered devices, we used methods with low computational complexity. First, we avoided complex features for ML. Second, the high correlation between the features allowed reducing the feature dimensionality (and complexity) by 34% (from 53 features to 35) while keeping the classifier performance. Third, we used a relatively simple FD method, for which we previously showed that it is comparable to more complex methods using ML [44,48].

We used the first dataset to perform an exhaustive feature analysis by first extracting 53 features and then selecting the subset with the relevant ones. The same experimental setup was used for each of the other datasets too, and this way we avoided the problem of tuning parameters to a particular dataset. In particular, we tested five machine learning algorithms (J48, NB, SVM, RF, and KNN) on each of the datasets. The conclusion is that the RF achieves the best performance for each dataset and almost any sensor location.

Table 12 summarizes the AR experiments (*i.e*., it shows the accuracy for the available sensor locations for each dataset). On the first dataset (JSI) we compared the AR performance of 10 sensor locations. Even though the related work shows that with a wrist-worn device one should expect worse accuracy compared to devices worn on other body locations, our results showed that the left wrist achieves 72% accuracy, which is better compared to the right one (68%), and also better compared to the elbow and the chest (67%), but worse compared to the ankle, knee, and belt (77%). These findings were confirmed on the second dataset. On the third (Opportunity) dataset our method outperformed the related work, indicating that our feature-preprocessing (feature extraction and feature selection) creates better input data. Finally, on a real-life unlabeled dataset the recognized activities captured the subject’s daily rhythm and activity level.

Table 13 shows the number of false positives for the available sensor locations for each dataset. It can be noted that the number of the wrist false positives on the first dataset is comparable and even smaller (better) than most of the other locations. For the second dataset nine false positives were detected, which is better than the ankle and worse than the thigh and the chest. On the Opportunity dataset only one false positive was detected. Finally, the real-life dataset (57 days of data) showed that only nine false positives were detected. These results are encouraging given the fact that the wristband was worn by young and active people. The results show that using this method, one should not expect a lot of false alarms (one every five days).

The left *vs.* right analysis showed that the left hand, which was the non-dominant hand for all of our subjects (note that we do not have information about the three subjects in the Opportunity dataset), outperformed the right hand in both the AR and FD. The tests with the “switching wrists” (training the data on one wrist and testing on the other) showed that the accuracy drops, on average, by 25% for both wrists. However, when we trained the models on both wrists and applied it on each wrist individually, the accuracy increased, outperforming even the models that were trained and tested on the same wrist. Therefore, the best practical solution is to deploy a model trained on both wrists.

In all of the datasets, the subjects were young adults. This might be a problem if the fall detection system is meant to be used by users that need such system (*i.e*., elderly). This is a general problem with fall detection systems since it is almost impossible for the elderly to fall several times on the ground as real as possible. However, in the JSI dataset (the only one that includes falls), the volunteers were instructed how to act by a medical expert in order to mimic the elderly.

For future work we may use higher level features that provide information about the dependency of the instances [49]. Additionally, with the recent trends with Deep Learning, it would be interesting to apply Deep Learning for AR and FD on a wristband device. Some steps in this direction for AR are already made [20,21]. Next, beside the granular AR and fall detection, it would be a significant addition to the system to exploit energy expenditure estimation (calories burned, metabolic equivalent of a task—MET estimation). This way, the wristband device may be used by users that may benefit from such information, such as sportsmen (using a fitness tracker) or people that have diabetes problems [48]. Finally, we plan to implement the methods on an Android smartwatch and test the methods on data acquired over longer periods.

## Figures and Tables

**Table 1:** Summarization and statistics about the data in the four experimental datasets.

	JSI	FoS	Opportunity	Real-Life	Overall
**Number of subjects**	5	10	3	3	21
**Raw sensor data samples**	1,702,446	17,299,951	2,169,865	148,716,000	169,888,262
**#Instances (after applying sliding window)**	160,000	182,775	7200	2,460,000	2,809,975
**Average #instances per sensor**	16,000	45,694	3600	2,460,000	2,525,294
**Average #instances per person**	32,000	18,278	2400	820,000	872,678
**Average #instances per person per sensor**	3200	4569	1200	820,000	828,969
**Hours of data**	89	102	4	1367	1561
**Hours of data per sensor**	9	25	2	1367	1403
**Hours of data per wrist sensors**	18	25	4	1367	1414

**Table 2:** Overview of the extracted features. The number of features is represented with #.

Feature name	#	Feature name	#
Mean (X, Y, Z)	3	Quartile range (X, Y, Z)	3
Total mean	1	Coefficient of variation (X, Y, Z)	3
Area (X, Y, Z)	3	Absolute area (X, Y, Z)	3
Posture distance (X, Y, Z)	3	Total absolute area	1
Absolute mean (X, Y, Z)	3	Combined total absolute area	1
Variance (X, Y, Z)	3	Total magnitude	1
Skewness (X, Y, Z)	3	Mean crossing rate (X, Y, Z)	3
Kurtosis (X, Y, Z)	3	Correlation (1,2,3)	3
Quartiles 1-2-3 (X, Y, Z)	9	Amplitude (X, Y, Z)	3

**Table 3:** Random Forest (RF) confusion matrix and performance metrics (recall, precision, and F1 score) for left wrist from the JSI dataset. Overall # represents the total number of instances for the particular class.

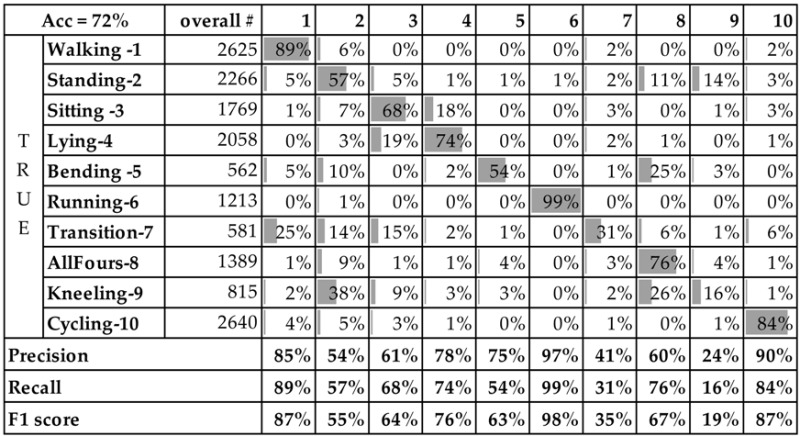

**Table 4:** RF confusion matrix and performance metrics (recall, precision and F1) for right wrist for FoS dataset. Overall # represents the total number of instances for the particular class.

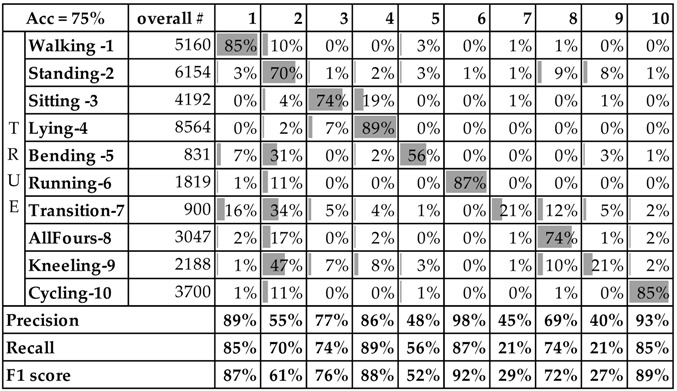

**Table 5:** RF confusion matrix and performance metrics (recall, precision and F1 score) for left wrist from the Opportunity dataset. Overall # represents the total number of instances for the particular class.

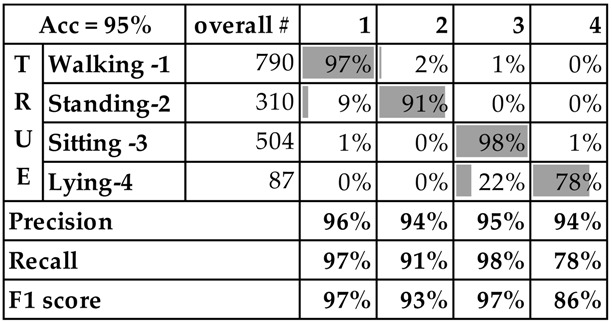

**Table 6:** Percentages of recognized lying activities for marked sleeping events.

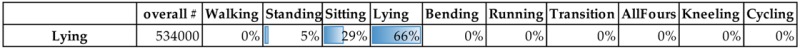

**Table 7:** Percentages of recognized lying activities for marked sleeping events.

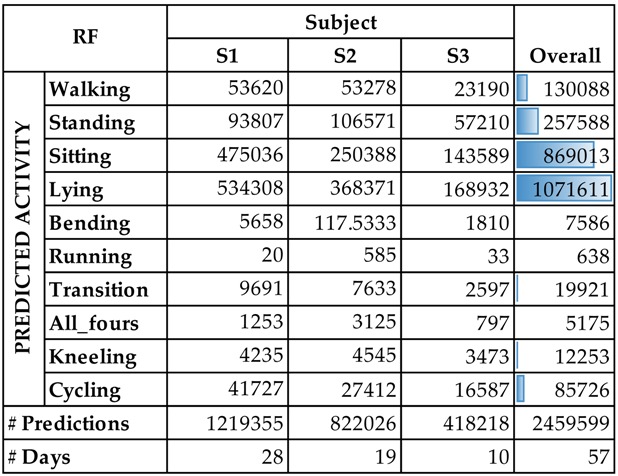

**Table 8:** Accuracy of fall detection (FD) for different sensor placements on the JSI dataset.

Metrics	Wris L	Wris R	Ankl L	Ankl R	Thigh L	Thigh R	Elbow L	Elbo R	Chest	Belt
TPR	70%	60%	33%	43%	53%	57%	57%	43%	47%	63%
TNR	90%	88%	86%	94%	83%	87%	84%	79%	88%	80%
Accuracy	85%	80%	76%	76%	82%	78%	82%	78%	74%	71%

**Table 9:** Fall Detection performance for the left wrist FD performance for the left wrist on the JSI dataset. Comparison of two methods: (AFP + Movement) and (AFP). AFP, Acceleration Fall Pattern.

		AFP + Movement Wrist Left (Non-Dominant)		AFP Wrist Left (Non-Dominant)	
		DETECTED		DETECTED	
		FALL	NON-FALL	FALL	NON-FALL	
TRUE	FALL	21	9	21	9	
	NON-FALL	10	90	63	37	
	TPR	0.70		0.70	
	TNR	0.90		0.37	
	Accuracy	0.85		0.45	

**Table 10:** Fall Detection performance for the right wrist on the JSI dataset. Comparison of two methods: (AFP + Movement) and (AFP).

		AFP + Movement Wrist Right (Dominant)		AFP Wrist Right (Dominant)	
		DETECTED		DETECTED	
		FALL	NON-FALL	FALL	NON-FALL
TRUE	FALL	17	13	19	11
	NON-FALL	13	87	55	45
	TPR	0.57		0.63	
	TNR	0.87		0.45	
	Accuracy	0.80		0.49	

**Table 11:** The recognition rate (recall) for each of the 10 activities and the overall accuracy for four combinations (Train-Test): Left-Left, Right-Right, Left-Right, Right-Left.

	Train-Test					
	Left-Left	Right-Right	Left-Right	Right-Left	(Left + Right)-Left	(Left + Right)-Right
Walking	89%	87%	82%	77%	90%	89%
Standing	57%	53%	8%	8%	58%	53%
Sitting	68%	67%	10%	20%	67%	62%
Lying	74%	75%	88%	85%	75%	75%
Bending	54%	51%	82%	26%	61%	56%
Running	99%	81%	78%	74%	99%	93%
Transition	31%	34%	49%	47%	29%	31%
All fours	76%	79%	70%	90%	78%	79%
Kneeling	16%	20%	0%	0%	17%	20%
Cycling	84%	74%	66%	62%	84%	76%
Accuracy	72%	68%	55%	53%	**73%**	**69%**

**Table 12:** Summary of the AR experiments—the accuracy of the available sensor locations.

Data	W_R	W_L	A_R	A_L	T_R	T_L	E_R	E_L	C	B
JSI	−4%	72%	+1%	+3%	−2%	+3%	+1%	0%	−3%	+5%
Faculty of Sports	76%		+7%		+7%				−4%	
Opportunity	−16%	77%								

**Table 13:** Summary of the FD experiments—the number of false positives (the bigger the number the worse the performance) of the available sensor locations.

Data	W_R	W_L	A_R	A_L	T_R	T_L	E_R	E_L	C	B
JSI	10	12	14	6	17	13	16	21	12	20
Faculty of Sports	9		16		2				1	
Opportunity	1	0								
